# CXCL5/NF-*κ*B Pathway as a Therapeutic Target in Hepatocellular Carcinoma Treatment

**DOI:** 10.1155/2021/9919494

**Published:** 2021-05-31

**Authors:** Xingqing Jia, Shuangqin Wei, Wujun Xiong

**Affiliations:** ^1^Department of Hepatology, Shanghai East Hospital, Tongji University School of Medicine, Shanghai 201399, China; ^2^Department of Gastroenterology, Shanghai Pudong Hospital, Fudan University Pudong Medical Center, Shanghai 201399, China

## Abstract

**Background:**

Hepatocellular carcinoma (HCC) is a common malignant cancer worldwide. CXCL5 has a role in inhibiting cell viability and metastasis in many tumors. In the present study, we investigated the role of CXCL5 in HCC and explored the underlying mechanism. *Material and Methods*. RT-qPCR and western blot were performed to evaluate the mRNA and protein levels of CXCL5. CCK-8 and transwell assay were applied to measure the proliferative and invasive abilities. Meanwhile, the Kaplan–Meier method was used to assess the survival of HCC patients.

**Results:**

CXCL5 was upregulated in HCC tissues, which predicted a shorter overall survival in HCC. CXCL5 was a target gene of miR-577, and its expression was mediated by miR-577 in HCC. Knockdown of CXCL5 suppressed HuH-7 cell proliferation, invasion, and EMT and inhibited the NF-*κ*B signaling pathway in cells. Moreover, knockdown of CXCL5 inhibited the xenograft growth of HuH-7 cells.

**Conclusion:**

Overexpression of CXCL5 predicts poor prognosis in HCC patients. Knockdown of CXCL5 inhibits cell proliferation and invasion through the NF-*κ*B signaling pathway in HCC. The newly identified role of the CXCL5/miR-577/NF-*κ*B axis provides novel insights into the targeted therapy of HCC.

## 1. Introduction

Hepatocellular carcinoma (HCC) is a major cause of cancer death, especially in Africa and Asia [1, 2]. Due to the hepatitis C virus epidemic, the incidence of HCC is increasing in Western countries [3]. The current treatment for HCC is limited to surgical resection, but resection results in a recurrence rate of more than 70% within 5 years, while about 80% are not suitable for surgery [4]. Therefore, it is urgent to explore potential biomarkers for the treatment.

C-X-C motif chemokine ligand 5 (CXCL5), known as ENA-78 or SCYB5, is a member of the CXC subfamily of chemokines; it binds the G-protein-coupled receptor chemokine (C-X-C motif) receptor-2 to recruit neutrophils, to promote angiogenesis and to remodel connective tissues [5]. CXCL5 was thought to play roles in cell proliferation, migration, and invasion of cancer [6, 7]. CXCL5 citrullination may exert inflammatory properties by recruiting monocytes to inflamed joint tissue in a mouse model of inflammatory arthritis [8]. CXCL5 acted as an important angiogenic factor in idiopathic pulmonary fibrosis and non-small-cell lung cancer [9, 10]. CXCL5 was involved in the interaction between cholangiocarcinoma cells and cancer-associated fibroblasts and inhibition of tumor-stromal interactions [7]. However, few studies have elucidated the roles of CXCL5 in HCC. Thus, the experiments were performed to explore the vital functions of CXCL5 in HCC.

The discovery of microRNAs (miRNAs) initiates a new generation of cognition in HCC [11, 12]. miRNAs negatively mediate gene expression through translational repression or mRNA degradation to be involved in tumor development [13]. According to a few reports, several miRNAs, including miR-122, miR-325, miR-206, miR-122, and miR-224 played crucial roles in HCC [14–17]. miR-577 acts as a tumor suppressor to suppress tumor growth and enhances chemosensitivity in colorectal cancer [18]. According to a previous study, miR-577 regulated cell proliferation and promoted G1-S phase transition in esophageal squamous cell carcinoma [19]. Similarly, miR-577 inhibited pancreatic *β*-cell function and survival in pediatric diabetes [20]. Wang et al. reported that, in non-small-cell lung cancer, miR-577 suppressed cell growth and EMT in regulating WNT2B via the Wnt/*β*-catenin pathway [21]. We observed that CXCL5 promoted HCC growth, cell invasion, the EMT, and the NF-*κ*B pathway in HCC. The expression of CXCL5 was regulated by miR-577 via directly targeting its 3'-UTR of mRNA.

## 2. Materials and Methods

### 2.1. Clinical Specimens

Pairs of HCC tissues and adjacent tissues were collected from 48 HCC patients at Shanghai East Hospital affiliated to Tongji University School of Medicine, Shanghai, China, from January 2016 to February 2021. Specimens were immediately frozen in liquid nitrogen and then stored at −80°C after surgery. All patients provided written informed consent, and the ethics committee of Shanghai East Hospital affiliated to Tongji University School of Medicine approved this study.

### 2.2. Cell Culture

HCC cells HuH-7 and a normal hepatocyte cell L-02 were purchased from the American Type Culture Collection (ATCC; Rockville, MD, USA). All cells were incubated in the DMEM medium (Invitrogen, Carlsbad, CA, USA) with 10% FBS (Sigma-Aldrich, Louis, MO, USA) at 37°C in a humidified chamber with 5 % CO_2_.

### 2.3. Transfection

The specific plasmids of shRNA-CXCL5 and their negative control were designed and synthesized at Gene-Pharma (Shanghai, China). HuH-7 cells were transfected and incubated in a 6-well plate. The Lipofectamine 2000 Reagent (Invitrogen, USA) diluted using an Opti-MEM/Reduced serum medium (Thermo Scientific, Shanghai, China) was used to perform the transfection. Geneticin (G418; Thermo Scientific, Shanghai, China) was used to select the stable transfection cells, while the transient transfection cells were harvested after transfected for 48 h.

### 2.4. Quantitative Real-Time PCR

The TRIzol Reagent (Invitrogen) and miRNeasy Mini Kit (Qiagen, Hilden, Germany) were employed to extract total mRNAs and miRNAs from tissues or cells. The Omniscript Reverse Transcription Kit (Qiagen) and TaqMan miRNA Reverse Transcription Kit (Applied Biosystems, Foster City, CA, USA) were used for synthesizing the first cDNA chain; the QuantiTect SYBR Green PCR Kit (Qiagen) and miRNA-specific TaqMan miRNA Assay Kit (Applied Biosystems) were used to carry out the qPCR in a Quantitect SYBR green PCR system (Qiagen). The relative levels of mRNA and miRNA were derived using a 2^−ΔΔCt^ method, and the GAPDH and U6 small nuclear RNA were utilized for normalization. The primers used for RT-qPCR were as follows: CXCL5 forward 5'-AGCTGCGTTGCGTTTGTTTAC-3', reverse 5'-TGGCGAACACTTGCAGATTAC-3'; GAPDH forward 5'-AAGGTGAAGGTCGGAGTCAA-3', reverse 5'-AATGAAGGGGTCATTGATGG-3'; miR-577 forward 5'-TGCGGTAGATAAAATATTGG-3', reverse 5'-GTGCAGGGTCCGAGGT-3'; and U6 forward 5'-GCTTCGGCAGCACATATACTAAAAT-3', reverse 5'-CGCTTCACGAATTTGCGTGTCAT-3'.

### 2.5. Western Blot Analysis

The total proteins were lysed by RIPA Lysis Buffer (Sigma, USA) containing 10% PMSF (Sigma). SDS-PAGE was applied to separate the protein, and then, the blots were electrotransferred to PVDF membranes (Millipore, USA). After being blocked by 5% fat-free milk at room temperature for 1 h, the membranes were incubated with primary antibodies, such as CXCL5 (1 : 1000; Abcam, Cambridge, USA), E-cadherin (1 : 1000; Abcam), N-cadherin (1 : 1000; Abcam), Vimentin (1 : 1000; Abcam), p-P65 (1 : 1000, Abcam), and P65 (1 : 1000, Abcam). Next, the blots were incubated by a secondary anti-rabbit HRP-conjugated antibody (Cell Signaling). The protein signals were captured using an Enhanced Chemiluminescence Detection Kit (ECL, Pharmacia Biotech, Arlington, USA).

### 2.6. MTT Assay

The HuH-7 cells were plated into 96-well plates and cultivated for 24-, 48-, 72-, and 96 h. Total 20 *μ*l of MTT (5 mg/ml, Sigma) was added into each well for 6 h of incubation. Next, the supernatant was discarded and 100 *μ*l of DMSO (Sigma) was added to each well. After agitating for 10 min, the absorbance at a wavelength of 570 nm was evaluated using an ELISA reader (Bio-Rad, Hercules, CA, USA).

### 2.7. Transwell Assay

The transwell insert (8 *μ*m membrane, Corning, Cambridge, MA) was placed in a 24-well plate to evaluate the cell invasive ability. The HuH-7 cells were suspended by the FBS-free RPMI-1640 medium and 200 *μ*l was added in the upper chamber, whereas the lower chamber was filled with 500 *μ*l medium containing 15% FBS, which acted as an inducer. After the cells were incubated for 24 h at 37°C, the noninvasive cells on the upper surface were removed by using cotton swabs. The invasive cells were fixed and stained using 4% paraformaldehyde and 10% crystal violet, respectively, and the cells were counted under a microscope (Olympus Corporation, Tokyo, Japan).

### 2.8. miRNA Target Prediction and Dual-Luciferase Reporter Assay

TargetScan was used to perform the prediction of target genes of miR-577, and we discovered that CXCL5 was one of the potential target genes. The binding sequence of UUUAUCU and AAAUAGA was mutated to confirm that miR-577 could not bind to the 3-UTR of CXCL5 mRNA in HCC cells. The wild type and the mutational 3'-UTR of CXCL5 were inserted into the dual-luciferase reporter vectors, which were named WT or MUT. The Lipofectamine 2000 Reagent (Invitrogen, USA) was used to cotransfect miR-577 mimic and WT or MUT vector into HuH-7 cells. Finally, the luciferase activity was measured using a dual-luciferase reporter assay system (Promega, USA).

### 2.9. Xenograft Assay in Nude Mice

All animal procedures were performed in accordance with protocols approved by the Institute Research Ethics Committee at Shanghai East Hospital affiliated to Tongji University School of Medicine, Shanghai, China. For the xenograft assay, 2 × 10^6^ HuH-7 cells transfected with shRNA-CXCL5 or negative control were subcutaneously injected into the left armpit of 5-week-old BALB/C nude mice. Tumor volumes and body weights were measured every 3 days, and tumor volumes were calculated using the formula volume = 0.5 × tumor length × tumor width^2^. After 26 days, CO^2^ was utilized for lethal anesthesia in mice; then, the weights of tumor were measured.

### 2.10. Statistical Analysis

All the statistical analyses were performed using SPSS 16.0 software (IBM, Armonk, NY, USA), and the data were presented as mean ± SD. Student's t test was performed to compare the differences between two groups; besides, one-way ANOVA was utilized to compare the differences between three or more groups. The association between CXCL5 expression and the overall survival for HCC patients was assessed by the Kaplan–Meier curve and log-rank test. *P* < 0.05 was considered to be statistically significant.

## 3. Results

### 3.1. Upregulation of CXCL5 Predicts Poor Prognosis of HCC Patients

**Figure 1:**
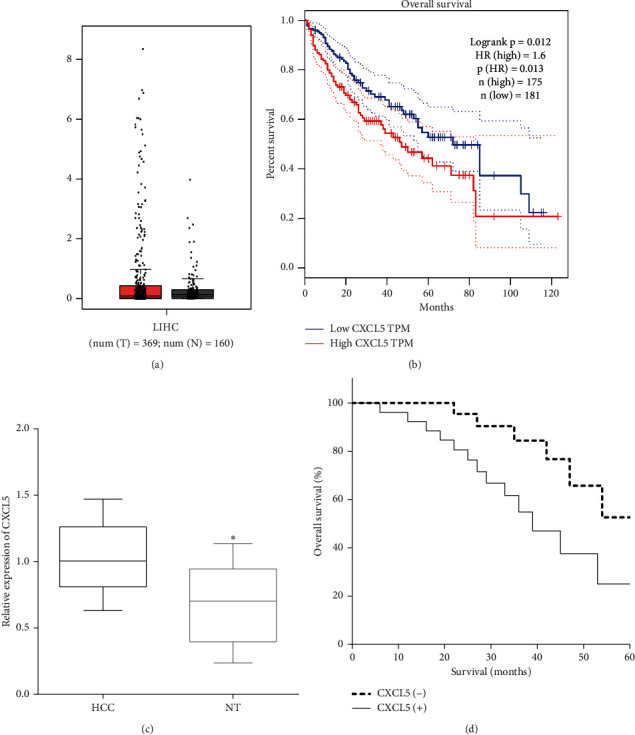
Upregulation of CXCL5 predicts poor prognosis of HCC. (a) The expression of CXCL5 in HCC tissues and normal tissues was detected in the GEPIA database. T: tumor tissues; N: normal tissues. (b) The GEPIA database showed that overexpression of CXCL5 predicted poor prognosis. (c) CXCL5 was less expressed in HCC tissues versus the corresponding peritumoral normal tissues vs. HCC, ^*∗*^*P* < 0.05. (d) Upregulation of CXCL5 was associated with a poor 5-year survival of HCC patients.

The expression of CXCL5 in HCC tissues and normal tissues was detected in the GEPIA database; although the expression of CXCL5 has no significance in HCC tissues than in normal tissues (Figure 1(a)), we observed that the overexpression of CXCL5 in HCC patients predicted poor prognosis (*P* < 0.05) (Figure 1(b)). In this study, the mRNA level of CXCL5 was assessed in 48 pairs of HCC and adjacent normal tissue, and we found that the expression of CXCL5 was overexpressed in HCC tissues as compared to adjacent normal tissue (*P* < 0.05) (Figure 1(c)). The Kaplan–Meier method elucidated that the expression of CXCL5 was associated with poor overall survival of HCC patients (*P*=0.0208) (Figure 1(d)).

### 3.2. Knockdown of CXCL5 Inhibits Cell Invasion, the EMT, and the NF-*κ*B Signal Pathway in HuH-7 Cells

**Figure 2:**
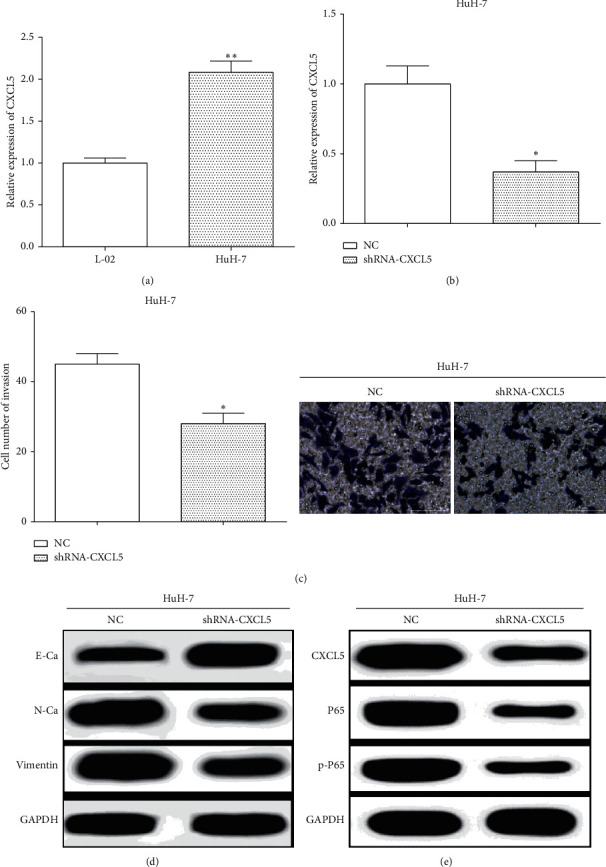
Knockdown of CXCL5 inhibits cell invasion, the EMT, and the NF-*κ*B signal pathway in HuH-7 cells. (a) The expressions of CXCL5 in L-02 and HuH-7 cells were measured vs. L-02, ^*∗∗*^*P* < 0.01. (b) The transfection efficiency of transfecting shRNA-CXCL5 in HuH-7 cells was calculated by RT-qPCR vs. NC group, ^*∗*^*P* < 0.05. (c) The invasive ability after changing the expression of CXCL5 in HuH-7 cells was measured vs. NC, ^*∗*^*P* < 0.05. (d) The levels of EMT-associated proteins were assessed by western blot in HuH-7 cells. (e) The expressions of P65 and p-P65 in HuH-7 cells were calculated in HuH-7 cells.

The expressions of CXCL5 were evaluated in HCC cells HuH-7 and a hepatocyte cell L-02. Moreover, we found that the expression of CXCL5 was lower in L-02 cells than that in HuH-7 cells (*P* < 0.01) (Figure 2(a)). To assess the roles of CXCL5, shRNA-CXCL5 was employed to downregulate CXCL5 in HuH-7 cells, and the transfection efficiency was calculated by RT-qPCR (Figure 2(b)). Transwell assay was utilized to measure the invasive ability after changing the expression of CXCL5 in HuH-7 cells, and we found that transfection of shRNA-CXCL5 enhanced the invasive ability (*P* < 0.05) (Figure 2(c)).

Moreover, the levels of EMT and pathway-associated proteins were assessed by western blot in HuH-7 cells. We found that knockdown of CXCL5 elevated the expression of E-cadherin, while suppressing N-cadherin and Vimentin expression in HuH-7 cells (Figure 2(d)), which suggested that knockdown of the CXCL5 suppressed the EMT. In addition, knockdown of CXCL5 inhibited the expression of P65 and p-P65 in HuH-7 cells (Figure 2(e)), which proved that knockdown of the CXCL5 inhibited the activation of the NF-*κ*B pathway. All the results revealed that knockdown of CXCL5 inhibited cell invasion, the EMT, and the NF-*κ*B signaling pathway.

### 3.3. Knockdown of CXCL5 Suppresses the Growth of HCC *In Vitro* and *In Vivo*

**Figure 3:**
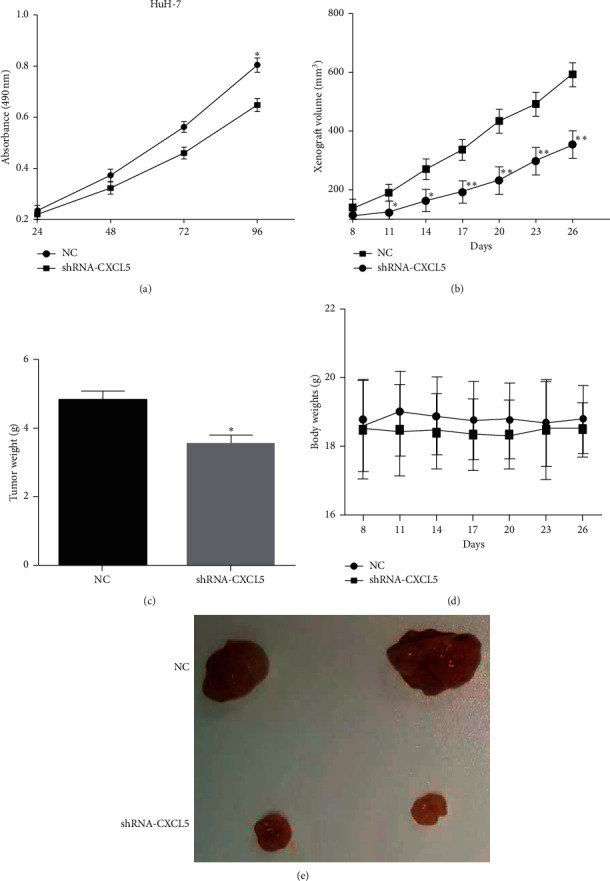
Knockdown of CXCL5 suppresses the growth of HCC *in vitro* and *in vivo*. (a) MTT assay was used to measure cell proliferation after silencing CXCL5 in HuH-7 cells vs. NC, ^*∗*^*P* < 0.05. (b) The volumes of xenograft tumors were measured every 3 days in the group of transfecting shRNA-CXCL5 and the control group vs. NC, ^*∗*^*P* < 0.05. (c) The weights of xenograft were calculated in the shRNA-CXCL5 and control group. vs. NC, ^*∗*^*P* < 0.05. (d) The body weights of mice were measured in the shRNA-CXCL5 and control group. (e) The picture of the tumor in the shRNA-CXCL5 and control group.

MTT assay was used to measure cell proliferation after knocking down CXCL5 in HuH-7 cells, and the results illuminated that transfection of shRNA-CXCL5 inhibited cell proliferative ability (*P* < 0.05) (Figure 3(a)). To further explore the functions of CXCL5 *in vivo*, HuH-7 cells transfected with shRNA-CXCL5 were used to perform tumor formation in nude mice. The HuH-7 cells that stably transfected shRNA-CXCL5 or control plasmid were subcutaneously injected into the nude mice. The volumes of xenograft tumors were measured every 3 days, and the group of transfecting shRNA-CXCL5 had a lower growth rate than the control group, which indicated that silencing of CXCL5 inhibited the HCC growth *in vivo* (Figure 3(b)). After 26 days, the nude mice were sacrificed. The weights of xenograft were calculated, and the tumor weights of the CXCL5 knockdown group were lower than that of the control group (*P* < 0.05) (Figure 3(c)), whereas the body weights were not significant between the CXCL5 knockdown group and control group (Figure 3(d)). The morphology of the xenograft is shown in Figure 3(e). All the findings indicated that silencing of CXCL5 inhibited the growth of HCC *in vitro* and *in vivo*.

### 3.4. CXCL5 Is a Target Gene of miR-577

**Figure 4:**
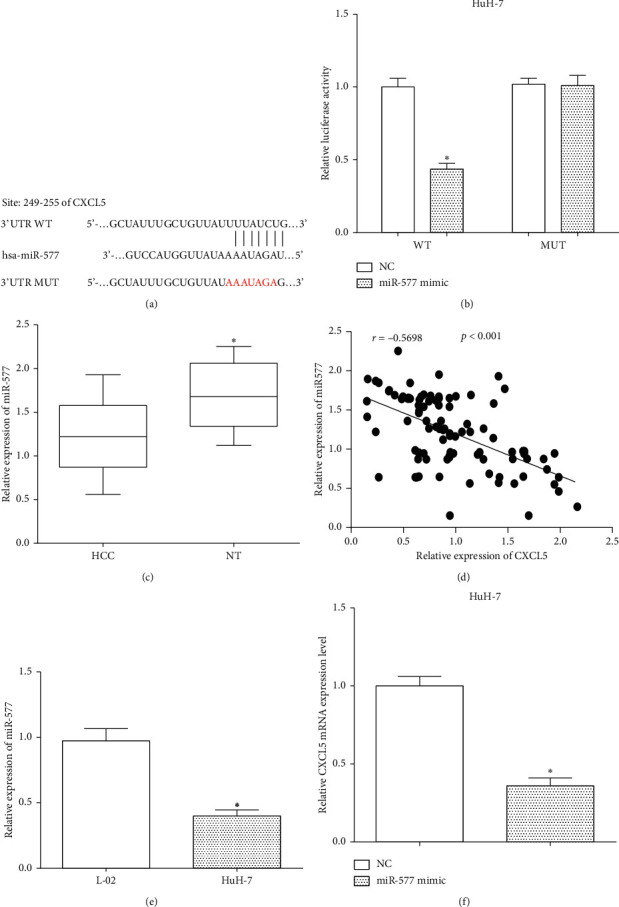
CXCL5 is a target gene of miR-577. (a) CXCL5 was predicted to be a target gene of miR-577 using TargetScan. (b) The luciferase activity was calculated using the luciferase reporter assay vs. NC, ^*∗*^*P* < 0.05. (c) RT-qPCR was employed to assess the expression of miR-577 in tissues vs. HCC, ^*∗*^*P* < 0.05. (d) The correlation analysis between CXCL5 and mir-577 in tissues. (e) The expression of miR-577 was measured in the HCC cell line HuH-7 and hepatocyte cell line L-02 vs. L-02, ^*∗*^*P* < 0.05. (f) The mRNA levels of CXCL5 were evaluated after transfecting miR-577 mimic in HuH-7 cells vs. NC, ^*∗*^*P* < 0.05.

CXCL5 was predicted to be a target gene of miR-577 using TargetScan, and the binding site was located at 249 to 255 on the 3'-UTR of CXCL5 mRNA. The potential binding sites were mutated to validate whether miR-577 directly binds to the potential binding site of CXCL5 (Figure 4(a)). Furthermore, the luciferase reporter assay results proved that miR-577 reduced the luciferase activity of HuH-7 cells that were transfected with wild-type CXCL5 3'-UTR (*P* < 0.05); however, it makes no difference on the luciferase activity of cells transfected with mutated CXCL5 3'-UTR (*P* > 0.05) (Figure 4(b)). RT-qPCR was employed to assess the expression of miR-577 in tissues and cells. Moreover, we found that the expression of miR-577 in HCC tissues was lower than that in peritumoral normal tissues (*P* < 0.05) (Figure 4(c)). Correlation analysis showed that the expression of CXCL5 was negatively correlated with miR-577 in tissues (Figure 4(d)). Similarly, miR-577 was less expressed in the HCC cell line HuH-7 compared with the hepatocyte cell line L-02 (*P* < 0.05) (Figure 4(e)). Moreover, the mRNA levels of CXCL5 were evaluated after transfecting miR-577 mimic in HuH-7 cells, and it showed that overexpression of miR-577 inhibited the expression of CXCL5 in HuH-7 cells (*P* < 0.05) (Figure 4(f)). All the results indicated that miR-577 regulated the expression of CXCL5 via directly targeting its 3'-UTR of mRNA in HCC cells HuH-7.

## 4. Discussion

HCC is one of the most common causes of cancer-related deaths worldwide, with a lower 5-year survival rate [22, 23]. However, the molecular mechanisms involved in HCC remain poorly understood.

CXCL5 acts as an oncogene and enhances cell growth and metastasis in several tumors, including bladder cancer, pancreatic cancer, cervical cancer, and cutaneous melanoma [24–27]. Previous studies reported that CXCL5 was overexpressed in the intestinal epithelium in inflammatory bowel disease and also in malignant pancreatic diseases [28, 29]. CXCL5 directly enhances tumor cell survival and proliferation in gastric cancer [30]. Consistent with the previous studies, we observed that CXCL5 was upregulated in HCC tissues compared to the normal tissues. Overexpression of CXCL5 was associated with the poor prognosis of HCC patients. Consistent with all the findings, we proposed that CXCL5 was upregulated in the HCC cell line HuH-7 compared with the normal cell line L-02. Moreover, knockdown of CXCL5 inhibited cell invasiveness and the EMT abilities in the HCC line HuH-7. In colorectal cancer, CXCL5 promoted tumor angiogenesis via the AKT/NF-*κ*B pathway [31]. We also revealed that knockdown of CXCL5 inhibited the NF-*κ*B signaling pathway.

Numerous studies have shown that miRNAs were associated with translational repression and mRNA degradation at the posttranscriptional level [32, 33]. Xue et al. reported that miR-577 acted as a tumor suppressor to inhibit cell proliferation, migration, and invasion in papillary thyroid carcinoma [34]. Several reports showed that MiR-577 suppressed metastasis and EMT of breast cancer [35]. In addition, miR-577 suppressed tumor growth of HCC, which was consistent with the findings in glioblastoma [36]. It is the first time to propose that CXCL5 was a target gene of miR-577 in HCC.

## 5. Conclusions

Overexpression of CXCL5 predicts poor prognosis in HCC patients. Also, knockdown of CXCL5 impaired cell invasion and EMT via the NF-*κ*B signaling pathway in HuH-7 cells. CXCL5 was a target gene of miR-577, and its expression was regulated by miR-577 in HCC. The newly identified role of the CXCL5/miR-577/NF-*κ*B axis provides novel insights into the targeted therapy of HCC.

## Data Availability

The data used to support the findings of this study are available from the corresponding author upon request.
